# 

**Published:** 1916-07-08

**Authors:** 


### CONTENTS.

| | PAGE |
|---|---|
| **Some Disabilities from War Injuries** ... ... | 337 |
| **Mixed Classes in Medical Education** ... ... | 338 |
| **Hospital and Institutional News** ... ... ... | 339 |
| The Royal Medical Benevolent Fund—The Temple Fête—Sir W. Osler on the Welsh Medical School—The Ideal Dean for the Welsh Medical School—The Late Lord Sandwich—Hospital Secretary's Son Killed—Educational Facilities for Women Students in London Hospitals—A Veteran of the Sikh Wars—Resignation of a Medical Superintendent—Chairman's Gift to Macclesfield Infirmary—A Masonic War Hospital—Textile Supplies for Hospitals—The Royal Victoria Infirmary, Newcastle-on-Tyne—Alleged Embezzlement of a Legacy to a Hospital—The War and the Out-Patient Department—The New Wing at the Royal Gwent Hospital, Newport—A Sphere of Usefulness for the Conscientious Objector—Great Northern Hospital's Diamond Jubilee—Garden Parties for the Wounded—A Dog on the Strength—Tribute to a Hospital Secretary—An Endowed Dispensary for Dublin—*Gazettes* for Sanatoria—A Welsh Bard in the R.A.M.C.—Consumption and the Boot Trade—A War Vacancy at Withington—Tuberculosis and the War—The Porter's Beer—This Week's Drug Market. | |
| **The Control of Cancer** ... ... ... ... ... | 345 |
| Two Books for Non-Medical Readers. | |
| **Practical Notes on Diagnosis and Treatment** | 346 |
| **Human Temperaments** ... ... ... ... ... | 347 |
| VI.—The Man of Business. | |
| **Guy's Hospital** ... ... ... ... ... ... ... | 348 |
| **Hospital Architecture and Construction** ... | 349 |
| South London Hospital for Women. | |
| **The Matrons' and Sisters' Department** ... | 351 |
| Economic Conditions in Nurse-Training. | |
| Round the Hospitals. | |
| The Queen's Graciousness—Training in Small Institutions—Probationers of the Best—How to Secure Them—Mrs. Bedford Fenwick's Claim. | |
| **Textile Supplies for Hospitals** ... ... ... | 353 |
| XIII.—The Care of Textiles. | |
| **The Editor's Letter-Box** ... ... ... ... ... | 355 |
| Nurse Training in War-Time—Necessitous Ladies' Holiday Fund. | |
| **Passing Events and Topics** ... ... ... ... | 355 |
| **The Convalescent Soldier** ... ... ... ... ... | 356 |
| Wakeswood Home, near Andover. | |
| **The Reduction of Infant Mortality** ... ... | 357 |
| The Priestley Lecture, by the Duchess of Marlborough. | |
| **The College of Nursing, Limited**... ... ... | 358 |
| Official Notices. | |
| **Parliament and the Profession** ... ... ... | vi |
| **Matrons' and Sisters' Appointments** ... ... | vi |
| **The Institutional Worker** ... ... (Suppl.) | 1 |
| Should Hospital "Letters" be Issued? : Answer to the June Question. | |
| Hints on Raising Funds. | |

